# Abuse Potential and Pharmacodynamic Characteristics of Oral and Intranasal Eluxadoline, a Mixed *μ*- and *κ*-Opioid Receptor Agonist and *δ*-Opioid Receptor Antagonist[Fn FN3]

**DOI:** 10.1124/jpet.116.236547

**Published:** 2016-12

**Authors:** N. Levy-Cooperman, G. McIntyre, L. Bonifacio, M. McDonnell, J. M. Davenport, P. S. Covington, L. S. Dove, E. M. Sellers

**Affiliations:** Altreos Research Partners, Inc., Toronto, Ontario, Canada (N.L.-C.); IntelliDev Consulting, LLC, Lansdale, Pennsylvania (G.M.); Lodestar Pharma Consulting, LLC, Durham, North Carolina (L.B.); INC Research Toronto, Inc. Early Phase CRO, Toronto, Ontario, Canada (M.M.); Furiex Pharmaceuticals, Inc., an affiliate of Allergan plc, Parsippany, New Jersey (J.M.D., P.S.C., L.S.D.); DL Global Partners Inc. and University of Toronto, Toronto, Ontario, Canada (E.M.S.)

## Abstract

Drugs with *μ*-opioid receptor (OR) activity can be associated with abuse and misuse. The peripherally acting mixed *μ*-OR and *κ*-OR agonist and *δ*-OR antagonist eluxadoline is approved in the United States for the treatment of irritable bowel syndrome with diarrhea. In two separate crossover studies, we evaluated the oral and intranasal abuse potential of eluxadoline versus placebo and the active control oxycodone. Healthy recreational opioid users received eluxadoline 100, 300, and 1000 mg, oxycodone 30 and 60 mg, and placebo (oral study), or eluxadoline 100 and 200 mg, oxycodone 15 and 30 mg, and placebos matched to eluxadoline and oxycodone (intranasal study). In the oral study, Drug Liking Visual Analog Scale (VAS) peak (maximum) effect (E_max_) score (primary endpoint) was significantly greater with eluxadoline 300 and 1000 mg versus placebo, but scores were significantly lower versus oxycodone. Following intranasal insufflation of eluxadoline, Drug Liking VAS E_max_ scores were not statistically different versus placebo, and were significantly lower versus oxycodone. Across other subjective measures, eluxadoline was generally similar to or disliked versus placebo. Pupillometry indicated no or minimal central effects with oral and intranasal eluxadoline, respectively. Adverse events of euphoric mood were reported with oral and intranasal eluxadoline but at a far lower frequency versus oxycodone. These data demonstrate that eluxadoline has less abuse potential than oxycodone in recreational opioid users.

## Introduction

The US Department of Justice Drug Enforcement Agency (DEA) assesses new drugs to determine if they should be scheduled under the Controlled Substances Act by analyzing eight factors: the actual or relative potential for abuse; scientific evidence of the drug’s pharmacological effects; the state of current scientific knowledge regarding the drug; its history and current pattern of abuse; the scope, duration, and significance of abuse; the risk, if any, to public health; its psychologic or physical dependence liability; and whether the substance is an immediate precursor to a substance already controlled (http://www.fda.gov/downloads/drugs/guidancecomplianceregulatoryinformation/guidances/ucm198650.pdf). Human abuse potential studies are often required as part of the abuse liability assessment, and the study design is influenced by a number of factors including the pharmacokinetics (PK), solubility, unit dose size, and potential routes of administration of the drug, along with historical ways in which drugs in the same class are abused.

Eluxadoline is a peripherally acting mixed *μ*-opioid receptor (OR) and *κ*-OR agonist and *δ*-OR antagonist with low oral bioavailability that is not subject to metabolism and is primarily excreted unchanged in the feces (Fujita et al., 2014; Davenport et al., 2015). The *δ*-OR activity of eluxadoline might mitigate the constipating effects of unopposed *μ*-OR agonism (Wade et al., 2012). Eluxadoline was well tolerated and demonstrated efficacy in treating the abdominal pain and diarrhea of irritable bowel syndrome with diarrhea (IBS-D) in two large, randomized, placebo-controlled Phase 3 trials (Lembo et al., 2016), and is currently approved in the US for the treatment of adults with IBS-D (http://www.accessdata.fda.gov/drugsatfda_docs/label/2015/206940s000lbl.pdf).

Centrally acting drugs with *μ*-OR activity, such as oxycodone, hydrocodone, and morphine, are often associated with abuse and misuse (Walsh et al., 2008; Stoops et al., 2010; Wightman et al., 2012). However, the peripherally acting antidiarrheal *μ*-OR agonist loperamide has been demonstrated to have relatively low abuse potential in humans, despite having positive reinforcing effects when administered intravenously to rhesus monkeys (Yanagita et al., 1979; Jaffe et al., 1980). Properties limiting the abuse potential of loperamide include its low solubility and poor oral bioavailability (http://www.accessdata.fda.gov/drugsatfda_docs/label/2005/017694s050lbl.pdf); however, recent reports suggest that illicit drug users, specifically those who are opioid-dependent, may use other drugs (e.g., CYP3A4, CYP2C8, and/or P-glycoprotein inhibitors) together with loperamide in an attempt to increase its absorption and penetration across the blood-brain barrier and enhance its euphoric effects (http://www.fda.gov/downloads/Drugs/DrugSafety/UCM505108.pdf).

In preclinical studies, eluxadoline was demonstrated to bind with high affinity to recombinantly expressed human *μ*-OR (K_i_: 1.7 nM) and with modest affinity to recombinant human *κ*-OR (K_i_: 55 nM) (Wade et al., 2012). Eluxadoline displayed a lower binding affinity for endogenous human *δ*-OR (K_i_: 367 nM) but bound to rat *δ*-OR with a K_i_ of 1.3 nM (Wade et al., 2012). In functional assays, eluxadoline is an agonist at *μ*-OR and *κ*-OR and an antagonist at *δ*-OR. Additionally, eluxadoline was demonstrated to have low oral bioavailability in rats and monkeys (Wade et al., 2012). In a toxicity study, monkeys treated with oral eluxadoline over 9 months did not show any behavioral changes indicative of central nervous system effects (US DEA, 2015b). Additionally, no behaviors suggestive of withdrawal were observed in either rats or monkeys during the observed recovery periods of various toxicity studies (US DEA, 2015b). However, as with loperamide, eluxadoline had positive reinforcing effects when administered intravenously to rhesus monkeys. Eluxadoline produced generalization to the morphine cue in a drug discrimination study at 10 mg/kg, and was self-administered to a greater extent than saline in heroin-trained monkeys at a dose of 3.2 mg/kg (US DEA, 2015b).

On the basis of the findings from the preclinical studies, the human abuse potential of eluxadoline was evaluated as part of the drug development program. Here we report the results of two studies that investigated the oral and intranasal abuse potential of therapeutic and supratherapeutic doses of eluxadoline in healthy recreational opioid users, compared with that of placebo and oxycodone, a Category II scheduled full *μ*-OR agonist.

## Materials and Methods

### 

#### Ethical Conduct of the Study.

Both studies complied with the principles of the Declaration of Helsinki and the International Conference on Harmonisation for Good Clinical Practice Guidelines. The studies were approved by the institutional review board of the participating study center, and participants gave their written informed consent prior to the screening assessments.

#### Study Design.

Both the oral and intranasal abuse potential studies were randomized, double-blind, double-dummy, placebo- and active-controlled, six-way crossover studies; however, the intranasal abuse potential study included two placebo controls matched to eluxadoline and oxycodone (Supplemental Fig. 1). The pretreatment phase of both studies included screening, a naloxone challenge test, and a qualification phase to determine if participants were suitable for entry into the treatment phase. Qualified participants were randomized to one of six treatment sequences, with a minimum washout of seven (oral study) or three (intranasal study) days between treatments.

#### Study Population.

For both studies, participants were healthy, nondependent, recreational opioid users, defined as having reported use of opioids for nontherapeutic purposes on ≥10 occasions within the past year, and ≥1 occasion in the 8 weeks prior to screening. For the intranasal study, participants were also required to have reported experience with intranasal drug administration, defined as ≥3 occasions of intranasal drug use within the past 12 months, and ≥1 occasion of intranasal opioid use during the 3 months prior to screening.

Participants who showed symptoms of withdrawal following the naloxone challenge test, who had a history or current diagnosis of substance dependence, or who had participated in, were participating in, or planned to seek treatment of substance-related disorders were excluded from both studies.

To participate in the treatment phase, volunteers had to meet the following criteria during the qualification phase: ability to distinguish oxycodone (oral: 40 mg; intranasal: 20 mg) from placebo on the Drug Liking Visual Analog Scale (VAS), defined as a ≥15-point increase in drug liking relative to placebo within 2 hours following oral or intranasal administration; an acceptable placebo response, defined as a Drug Liking VAS response of 45–55 (inclusive); and tolerability of study treatments (no episodes of vomiting within 2 hours postdose for both studies and no episodes of sneezing within 30 minutes postdose in the intranasal study).

#### Drug Selection and Doses.

In the oral study, participants received eluxadoline 100, 300, and 1000 mg, oxycodone hydrochloride immediate release (IR) 30 and 60 mg, and matching placebo tablets as controls, administered in a double-dummy fashion under fasting conditions. All dosing was completed within 5 minutes. In the intranasal study, participants received crushed eluxadoline 100 and 200 mg, and crushed oxycodone hydrochloride IR 15 and 30 mg. Owing to differences between oxycodone and eluxadoline in their weight and resulting bulk once crushed, two placebo arms were included: placebo lactose, weight matched to oxycodone, and placebo to match eluxadoline 200 mg. Eluxadoline 200 mg was selected as the maximal dose for the intranasal study because of the volume of the crushed tablets (1648 mg), with some evidence suggesting recreational drug users can insufflate a maximum of approximately 900–1000 mg of powder (Sellers and Spivey, 2009). Participants were instructed to intranasally self-administer crushed doses of study drug as quickly as possible, and within 5 minutes.

#### Measures and Endpoints.

The primary endpoint in both studies was Drug Liking VAS peak (maximum) effect (E_max_) (bipolar, 0: strong disliking, 50: neutral, 100: strong liking). Multiple additional subjective pharmacodynamic (PD) measures were assessed at predose for those measures not directly related to drug effects, and at intervals up to 24 hours postdose. Additional balance of effects measures were Overall Drug Liking VAS (bipolar, 0: strong disliking, 50: neutral, 100: strong liking), Take Drug Again VAS (bipolar, 0: definitely not, 50: neutral, 100: definitely so), and Subjective Drug Value. Positive effects measures were High VAS and Good Effects VAS (both unipolar, 0: definitely not, 100: definitely so), and Addiction Research Center Inventory (ARCI) Morphine Benzedrine Group scale. Negative effects measures were Bad Effects VAS (unipolar, 0: definitely not, 100: definitely so), and ARCI Lysergic Acid Diethylamide Group scale. Other drug effects measures were Any Effects VAS (unipolar, 0: definitely not, 100: definitely so), Alertness/Drowsiness VAS (bipolar, 0: very drowsy, 50: neutral and 100: very alert), and ARCI Phenobarbital-Chlorpromazine-Alcohol Group scale. Drug Similarity VAS (unipolar, 0: definitely not, 100: definitely so) and subject-rated nasal effects (intranasal study only) were also assessed. Pupillometry was assessed as an objective measure of central opioid effects in both studies.

In both studies, blood samples for PK analysis were collected predose and at intervals up to 24 hours postdose. Adverse events (AEs) occurring during the study period were recorded, as well as standard safety measures. In the intranasal study only, an observer-rated assessment of intranasal irritation and percentage of dose insufflated was also conducted.

#### Statistical Analysis.

The following analysis sets were used in the two studies: PD analysis set, defined as all participants in the randomized set who received ≥1 dose of study drug (oral study) or eluxadoline (intranasal study) in the treatment phase and had sufficient PD samples to allow accurate calculation of PD parameters; PK analysis set, defined as all participants in the randomized set who received ≥1 dose of eluxadoline in the treatment phase and had sufficient PK samples to allow accurate calculation of PK parameters; and safety analysis set, defined as all participants in the randomized set who received ≥1 dose of study drug in the treatment phase (oral study) or ≥1 dose of naloxone (intranasal study).

PK and PD data were summarized using descriptive statistics. PD endpoints were analyzed using a mixed-effects model for a crossover study. The model included treatment, period, sequence, and first-order carryover effect as fixed effects; baseline (predose) measurement as covariate where applicable; and subject nested within treatment sequence as a random effect. Residuals from the mixed-effects model were investigated for normality using the Shapiro-Wilk W test, and parameters were considered to be normally distributed if the probability value was >0.05. Parameters that did not meet the criteria were analyzed nonparametrically; pairwise treatment comparisons were assessed using the Wilcoxon signed-rank test on the within-subject differences.

## Results

### 

#### Participant Disposition, Demographics, and Baseline Characteristics.

In the oral abuse potential study, 58 participants were randomized to the naloxone challenge and qualification phase, 43 participants met qualification criteria, 40 were randomized to the treatment phase, and 33 completed the study. In the intranasal abuse potential study, 54 participants were randomized to the naloxone challenge/qualification phase, 39 participants qualified, 36 were randomized to the treatment phase, and 31 completed the study.

Participant demographics were similar for both studies (Table 1). Participants in both studies were predominantly male (oral study: 80.0%; intranasal study: 72.2%) and white (oral study: 72.5%; intranasal study: 83.3%). The mean age (±S.D.) was 39.8 ± 9.2 in the oral study, and 35.6 ± 8.8 in the intranasal study.

**TABLE 1:** Participant demographics and baseline characteristics: oral and intranasal abuse potential studies (randomized sets)

	Oral Abuse Potential Study (*n* = 40)	Intranasal Abuse Potential Study (*n* = 36)
Mean age, years (S.D.)	39.8 (9.2)	35.6 (8.8)
Male, *n* (%)	32 (80.0)	26 (72.2)
Race, *n* (%)		
White	29 (72.5)	30 (83.3)
Black or African American	7 (17.5)	5 (13.9)
Asian	3 (7.5)	1 (2.8)
American Indian or Alaska Native	1 (2.5)	–
Ethnicity, *n* (%)		
Hispanic or Latino	3 (7.5)	0 (0.0)
Not Hispanic or Latino	37 (92.5)	36 (100.0)
Mean BMI, kg/m^2^ (S.D.)	26.5 (3.2)	25.5 (3.0)
Prior recreational drug usage, *n* (%)		
Opioids and morphine derivatives	40 (100.0)	36 (100.0)
Cannabinoids	29 (72.5)	32 (88.9)
Stimulants	29 (72.5)	29 (77.8)
Depressants	8 (20.0)	16 (44.4)
Hallucinogens	8 (20.0)	15 (41.7)
Dissociative anesthetics	3 (7.5)	9 (25.0)
Number of times opioids used, mean (S.D.)		
In past 8 weeks	10.2 (6.1)	13.1 (11.8)
In past 12 months	54.8 (37.7)	67.6 (50.9)
Number of times intranasal opioids used, mean (S.D.)		
In past 3 months	—	11.9 (9.8)
In past 12 months	—	57.7 (51.4)

BMI, body mass index; S.D., standard deviation.

All participants in both studies reported prior use of opioids and morphine derivatives (Table 1), with the majority in the oral and intranasal studies, respectively, also reporting the use of cannabinoids (72.5%, 88.9%) and stimulants (72.5%, 77.8%). The mean numbers of instances of opioid usage in the 8 weeks prior to screening were 10.2 ± 6.1 and 13.1 ± 11.8 in the oral and intranasal studies, respectively, and in the past 12 months were 54.8 ± 37.7 and 67.6 ± 50.9, respectively. Additionally, participants in the intranasal study reported a mean number of 11.9 ± 9.8 instances of intranasal opioid use in the past 3 months, and 57.7 ± 51.4 in the past 12 months.

#### PD Balance of Effects Measures—Drug Liking VAS.

**Fig. 1.**
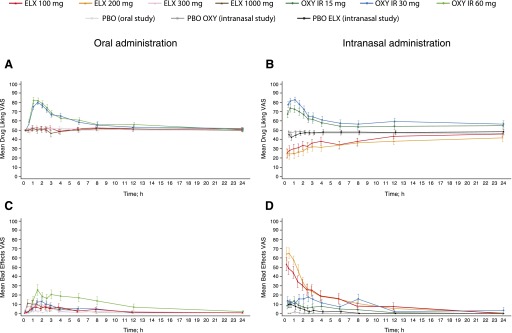
Subjective “at this moment” measures of drug effects following oral or intranasal drug administration. (A) Drug Liking VAS scores over 24 hours after oral dosing (mean ±S.E.). (B) Drug Liking VAS scores over 24 hours after intranasal dosing (mean ±S.E.). (C) Bad Effects VAS scores over 24 hours after oral dosing (mean ±S.E.). (D) Bad Effects VAS scores over 24 hours after intranasal dosing (mean ±S.E.). PD analysis sets. ELX, eluxadoline; IR, immediate release; OXY, oxycodone; PBO, placebo.

Mean Drug Liking VAS scores over 24 hours postdose are presented in Fig. 1A (oral study) and Fig. 1B (intranasal study).

In the oral study, the primary endpoint, Drug Liking VAS E_max_ score, indicating maximum “liking,” showed mean (±S.D.) scores of 85.8 ± 14.3 for oxycodone 30 mg, and 90.9 ± 11.5 for oxycodone 60 mg, which were 30–35 points higher than placebo, and median E_max_ scores were significantly higher with both doses of oxycodone compared with placebo (*P* < 0.0001 for both pairwise comparisons), demonstrating study validity (Table 2). Mean (±S.D.) E_max_ scores were close to neutral (50.0) following oral eluxadoline administration (100 mg: 56.8 ± 13.7; 300 mg: 58.7 ± 13.4; 1000 mg: 60.0 ± 14.8), with minimal mean increases (<5 points) with increasing eluxadoline dose (Table 2). Median differences compared with placebo were significant only for eluxadoline 300 and 1000 mg (*P* < 0.05 versus placebo for both doses). Mean E_max_ scores were between 25 and 35 points lower with eluxadoline compared with oxycodone, with median E_max_ scores being significantly higher for both doses of oxycodone compared with all eluxadoline doses (*P* < 0.0001 for all pairwise comparisons).

**TABLE 2:** Drug Liking VAS E_max_ and E_min_ following oral or intranasal drug administration (PD analysis sets)

**Oral Administration**							
Parameter	Statistic	PBO (*n* = 37)	OXY IR 30 mg (*n* = 37)	OXY IR 60 mg (*n* = 37)	ELX 100 mg (*n* = 35)	ELX 300 mg (*n* = 36)	ELX 1000 mg (*n* = 35)
Drug Liking VAS E_max_	Mean (S.D.)	54.3 (9.5)	85.8 (14.3)	90.9 (11.5)	56.8 (13.7)	58.7 (13.4)	60.0 (14.8)
	Median	51.0	88.0	100.0	51.0	51.5	51.0
	Median difference (IQR)						
	versus PBO	—	34.5 (23.5, 45.5) ***	41.0 (25.0, 49.0) ***	0.0 (–1.0, 1.0)	0.0 (0.0, 9.0) *	0.0 (0.0, 12.0) *
	versus OXY IR 30 mg	—	—	—	−35.0 (–45.0, –24.0) ***	−31.0 (–43.0, –17.0) **	−33.0 (–45.0, ­–16.0) **
	versus OXY IR 60 mg	—	—	—	−37.0 (–49.0, –26.0) ***	−33.0 (­–49.0, –20.0) ***	−35.0 (–49.0, ­–21.0) ***
Drug Liking VAS E_min_	Mean (S.D.)	46.5 (11.8)	48.6 (5.8)	42.5 (15.0)	45.0 (14.2)	44.9 (11.1)	38.3 (19.6)
	Median	50.0	50.0	50.0	50.0	50.0	50.0
	Median difference (IQR)						
	versus PBO	—	0.0 (0.0, 0.0)	0.0 (–1.0, 0.0)	0.0 (–1.0, 0.0)	0.0 (­–2.0, 0.0)	0.0 (­–14.0, 0.0) **
	versus OXY IR 30 mg	—	—	—	0.0 (–1.0, 0.0) *	0.0 (­–2.0, 0.0) *	0.0 (­–16.0, 0.0) **
	versus OXY IR 60 mg	—	—	—	0.0 (–1.0, 0.0)	0.0 (­–1.0, 0.0)	0.0 (­–16.0, 0.0)
**Intranasal Administration**							
Parameter	Statistic	PBO lactose (*n* = 32)	PBO ELX (*n* = 34)	OXY IR 15 mg (*n* = 32)	OXY IR 30 mg (*n* = 32)	ELX 100 mg (*n* = 32)	ELX 200 mg (*n* = 32)
Drug Liking VAS E_max_	Mean (S.D.)	49.1 (9.0)	52.2 (9.1)	79.5 (22.0)	88.7 (14.6)	53.2 (21.7)	54.9 (21.0)
	Median	51.0	51.0	81.0	95.0	51.0	51.0
	Median difference (IQR)						
	versus PBO lactose	—	0.0 (0.0, 0.0)	36.0 (16.5, 49.0) ***	47.0 (28.0, 49.0) ***	0.0 (–1.0, 2.0)	0.0 (–1.0, 20.0)
	versus PBO ELX	—	—	—	—	0.0 (–1.0, 1.0)	0.0 (0.0, 12.0)
	versus OXY IR 15 mg	—	—	—	—	−28.0 (–48.0, -12.0) ***	−26.0 (–48.0, -9.0) ***
	versus OXY IR 30 mg	—	—	—	—	−45.0 (–49.0, -26.0) ***	−42.0 (–49.0, -11.0) ***
Drug Liking VAS E_min_	Mean (S.D.)	45.3 (14.3)	38.1 (19.2)	48.1 (16.3)	45.6 (16.9)	16.4 (21.7)	12.4 (20.1)
	Median	50.0	49.0	50.0	50.0	0.0	0.0
	LS mean difference (S.E.)						
	versus PBO lactose	—	−6.7 (4.1)	2.7 (4.2)	0.1 (4.2)	−28.4 (4.2) ***	−33.0 (4.20) ***
	versus PBO ELX	—	—	—	—	−21.7 (4.1) ***	−26.3 (4.1) ***
	versus OXY IR 15 mg	—	—	—	—	−31.1 (4.2) ***	−35.7 (4.2) ***
	versus OXY IR 30 mg	—	—	—	—	−28.5 (4.2) ***	−33.1 (4.2) ***

ELX, eluxadoline; IQR, interquartile range; OXY IR, oxycodone immediate release; PBO, placebo.

* *P* < 0.05, **P < 0.01, and ***P < 0.0001.

Following intranasal administration, mean Drug Liking VAS E_max_ scores (± S.D.) were 79.5 ± 22.0 for oxycodone 15 mg, and 88.7 ± 14.6 for the 30-mg dose, which were 30–35 points greater than with both placebo controls (Table 2), and median E_max_ scores were significantly greater for both doses of oxycodone compared with both placebos (*P* < 0.0001 for all pairwise comparisons). Mean (±S.D.) E_max_ scores were within the neutral range for both eluxadoline 100 mg (53.2 ± 21.7) and 200 mg (54.9 ± 21.0), and median differences compared with both placebos were not statistically significant for either eluxadoline dose (Table 2). Mean E_max_ scores were again 30–35 points higher for oxycodone than for eluxadoline, and median E_max_ scores were significantly greater for both doses of oxycodone compared with both eluxadoline doses (*P* < 0.0001 for all pairwise comparisons).

Following oral administration, mean (±S.D.) Drug Liking VAS peak (minimum) effect (E_min_) scores, indicating maximum “disliking,” were below neutral for all treatments, including placebo (Table 2), with eluxadoline 1000 mg showing an approximately 8-point lower score than placebo (38.3 ± 19.6 versus 46.5 ± 11.7). Median scores were at neutral (50.0) for all treatments. Following intranasal administration, a markedly lower mean E_min_ score (±S.D.) was observed with both eluxadoline 100 mg (16.4 ± 21.7) and 200 mg (12.4 ± 20.1) compared with both placebo lactose (45.3 ± 14.3) and placebo eluxadoline (38.1 ± 19.2; Table 2). Least squares (LS) mean E_min_ scores were significantly lower with both eluxadoline doses compared with both doses of oxycodone and both placebo controls (*P* < 0.0001 for all pairwise comparisons), indicating significant disliking of intranasal eluxadoline.

#### PD Balance of Effects Measures—Overall Drug Liking VAS and Take Drug Again VAS.

**Fig. 2.**
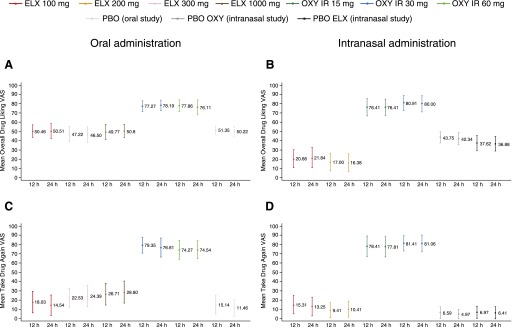
Subjective overall measures of drug effects following oral or intranasal drug administration. (A) Overall Drug Liking VAS scores at 12 and 24 hours after oral dosing (mean ± 95% CI). (B) Overall Drug Liking VAS scores at 12 and 24 hours after intranasal dosing (mean ±95% CI). (C) Take Drug Again VAS scores at 12 and 24 hours after oral dosing (mean ± 95% CI). (D) Take Drug Again VAS scores at 12 and 24 hours after intranasal dosing (mean ±95% CI). PD analysis sets. CI, confidence interval; ELX, eluxadoline; IR, immediate release; OXY, oxycodone; PBO, placebo.

Following oral administration, mean (±S.D.) scores at 12 hours were higher with both doses of oxycodone than with placebo for Overall Drug Liking VAS (30 mg: 77.3 ± 17.5; 60 mg: 77.9 ± 19.2) and Take Drug Again VAS (30 mg: 79.4 ± 25.7; 60 mg: 74.3 ± 30.5), with a significantly greater median score with oxycodone compared with placebo (*P* < 0.0001 for all pairwise comparisons) (Fig. 2, A and C, and Table 3). Mean (±S.D.) and median scores for Overall Drug Liking VAS with all doses of eluxadoline were neutral (100 mg: 50.5 ± 19.7; 300 mg: 47.2 ± 24.9; 1000 mg: 49.8 ± 24.0), with no significant difference in median scores between eluxadoline and placebo, and significantly greater median scores with oxycodone compared with eluxadoline (*P* < 0.0001 for all pairwise comparisons) (Fig. 2A and Table 3). Mean (±S.D.) Take Drug Again VAS scores were similar for eluxadoline 100 mg (18.0 ± 33.6) and placebo, and were higher with eluxadoline 300 and 1000 mg (300 mg: 22.5 ± 30.7; 1000 mg: 26.7 ± 34.1) compared with placebo (Fig. 2C and Table 3); median scores were approximately zero with all eluxadoline doses (100 mg: 0.0; 300 mg: 0.5; 1000 mg: 2.0) and with placebo, and median difference compared with placebo was significant only with eluxadoline 300 mg (*P* < 0.05). Median scores were significantly greater with oxycodone compared with eluxadoline (*P* < 0.0001 for all pairwise comparisons; Table 3).

**TABLE 3:** Effects on secondary measures following oral drug administration (PD analysis set)

Parameter	Statistic	PBO (*n* = 37)	OXY IR 30 mg (*n* = 37)	OXY IR 60 mg (*n* = 37)	ELX 100 mg (*n* = 35)	ELX 300 mg (*n* = 36)	ELX 1000 mg (*n* = 35)
Overall Drug Liking VAS							
At 12 h	Mean (S.D.)	51.4 (13.6)	77.3 (17.5)	77.9 (19.2)	50.5 (19.7)	47.2 (24.9)	49.8 (24.0)
	Median	50.0	81.0	78.0	50.0	50.0	50.0
	*P* value*^a^*	—	***	***	^††† ‡‡‡^	^††† ‡‡‡^	^††† ‡‡‡^
Take Drug Again VAS							
At 12 h	Mean (S.D.)	15.1 (30.9)	79.4 (25.7)	74.3 (30.5)	18.0 (33.6)	22.5 (30.7)	26.7 (34.1)
	Median	0.0	86.0	90.0	0.0	0.5	2.0
	*P* value*^a^*	—	***	***	^††† ‡‡‡^	* ^††† ‡‡‡^	^††† ‡‡‡^
Bad Effects VAS							
E_max_	Mean (S.D.)	8.8 (21.3)	23.4 (29.8)	40.5 (37.9)	13.3 (29.3)	26.9 (28.3)	23.1 (30.5)
	Median	0.0	3.0	28.0	0.0	14.5	11.0
	*P* value*^a^*	—	**	***	NS	**	*
Pupil diameter							
MPC	Mean (S.D.)	0.8 (0.7)	2.2 (0.7)	2.6 (0.8)	0.7 (0.5)	0.9 (0.5)	0.9 (0.5)
	Median	0.7	2.0	2.5	0.8	0.8	0.9
	*P* value*^a^*	—	***	***	^††† ‡‡‡^	^††† ‡‡‡^	^††† ‡‡‡^

ELX, eluxadoline; MPC, maximum pupil constriction; NS, not significant; OXY IR, oxycodone immediate release; PBO, placebo.

^a^ *P* values are for pairwise comparisons of median differences between treatments, assessed using the Wilcoxon signed-rank test.

* Significant versus PBO (*P* < 0.05); **significant versus PBO (*P* < 0.01);

*** significant versus PBO (*P* < 0.0001); ^†††^significant versus OXY IR 30 mg (*P* < 0.0001);

^‡‡^ significant versus OXY IR 60 mg (*P* < 0.01); ^‡‡‡^significant versus OXY IR 60 mg (*P* < 0.0001).

Mean (±S.D.) scores at 12 hours for both doses of intranasal oxycodone were higher than with placebo for Overall Drug Liking VAS (15 mg: 76.4 ± 25.3; 30 mg: 80.9 ± 22.8) and Take Drug Again VAS (78.4 ± 30.5; 81.4 ± 22.5), with significantly greater median scores for oxycodone compared with placebo (*P* < 0.0001 for all pairwise comparisons; Fig. 2, B and D, and Table 4). Mean (±S.D.) Overall Drug Liking scores were in the “disliking” range for intranasal eluxadoline (100 mg: 20.7 ± 26.9; 200 mg: 17.0 ± 26.7), with significantly lower LS mean scores for eluxadoline compared with both placebos and oxycodone (*P* < 0.0001 for all pairwise comparisons, except eluxadoline 100 mg compared with placebo eluxadoline (*P < 0.01; Table 4). Mean (±S.D.) Take Drug Again VAS scores were slightly greater with eluxadoline (100 mg: 15.3 ± 27.5; 200 mg: 9.4 ± 22.5) than with both placebos (Fig. 2D and Table 4); however, median scores with eluxadoline were not statistically significant compared with both placebos. Median scores with eluxadoline were significantly lower compared with oxycodone (*P* < 0.0001 for all pairwise comparisons; Table 4).

**TABLE 4:** Effects on secondary measures following intranasal drug administration (PD analysis set) Pairwise comparisons were assessed using the Wilcoxon signed-rank test.

Parameter	Statistic	PBO lactose (*n* = 32)	PBO ELX (*n* = 34)	OXY IR 15 mg (*n* = 32)	OXY IR 30 mg (*n* = 32)	ELX 100 mg (*n* = 32)	ELX 200 mg (*n* = 32)
Overall Drug Liking VAS							
At 12 h	Mean (S.D.)	43.8 (16.8)	37.6 (22.9)	76.4 (25.3)	80.9 (22.8)	20.7 (26.9)	17.0 (26.7)
	Median	50.0	50.0	76.0	88.0	6.5	0.0
	*P* value*^a^*	—	NS	***	***	*** ^§§ ††† ‡‡‡^	*** ^§§§ ††† ‡‡‡^
Take Drug Again VAS							
At 12 h	Mean (S.D.)	6.6 (17.7)	7.0 (19.0)	78.4 (30.5)	81.4 (22.5)	15.3 (27.5)	9.4 (22.5)
	Median	0.0	0.0	94.0	94.0	0.0	0.0
	*P* value*^b^*	—	NS	***	***	^††† ‡‡‡^	^††† ‡‡‡^
Bad Effects VAS							
E_max_	Mean (S.D.)	2.2 (8.8)	17.4 (25.0)	22.6 (28.2)	35.2 (35.0)	62.8 (38.7)	74.2 (32.0)
	Median	0.0	0.0	11.5	26.0	74.5	83.5
	*P* value*^b^*	—	*	*	***	*** ^§§§ ††† ‡‡^	*** ^§§§ ††† ‡‡‡^
Pupil diameter							
MPC	Mean (S.D.)	0.6 (0.5)	0.5 (0.5)	2.2 (0.8)	2.7 (0.8)	1.1 (0.6)	1.1 (0.6)
	Median	0.6	0.5	2.2	2.7	1.0	1.1
	*P* value*^a^*	—	—	***	***	*** ^§§§ ††† ‡‡‡^	*** ^§§§ ††† ‡‡‡^

ELX, eluxadoline; LS, least squares; MPC, maximum pupil constriction; NS, not significant; OXY IR, oxycodone immediate release; PBO, placebo.

^a^ *P* values are for pairwise comparisons of LS mean differences between treatments.

^b^ *P* values are for pairwise comparisons of median differences between treatments. Both assessed using the Wilcoxon signed-rank test.

* Significant versus PBO lactose (*P* < 0.05);***significant versus PBO lactose (*P* < 0.0001);^§§^significant versus PBO ELX (*P* < 0.01); ^§§§^significant versus PBO ELX (*P* < 0.0001); ^†††^significant versus OXY IR 15 mg (*P* < 0.0001); ^‡‡^significant versus OXY IR 30 mg (*P* < 0.01); ^‡‡‡^significant versus OXY IR 30 mg (*P* < 0.0001).

#### PD Negative Effects Measures.

Mean Bad Effects VAS scores following oral administration were relatively low with all treatments (Fig. 1C). Mean (±S.D.) scores were 15–30 points higher with oxycodone (30 mg: 23.4 ± 29.8; 60 mg: 40.5 ± 37.9) compared with placebo, with significantly higher median scores with oxycodone (30 mg, *P* < 0.01; 60 mg, *P* < 0.0001; Table 3). Mean Bad Effects VAS E_max_ scores (±S.D.) were greater with eluxadoline 300 mg (26.9 ± 28.3) than 100 mg (13.3 ± 29.3), and similar for 300 and 1000 mg (23.1 ± 30.5), with a significant median difference compared with placebo for eluxadoline 300 mg (*P* < 0.01) and 1000 mg (*P* < 0.05; Table 3). Mean Bad Effects VAS E_max_ scores were similar between oxycodone 30 mg and eluxadoline 300 and 1000 mg, with no significant median difference between oxycodone and eluxadoline for any dose (Table 3).

Following intranasal administration, eluxadoline was associated with increased scores on the Bad Effects VAS compared with other treatments until around 4 hours postdosing, with eluxadoline 200 mg showing scores slightly higher than the 100-mg dose. Oxycodone and placebo eluxadoline showed small increases (<20 points) from neutral until around 3 hours postdose, with oxycodone 30 mg showing higher scores compared with oxycodone 15 mg (Fig. 1D). Mean (±S.D.) E_max_ scores were 20–30 points greater with oxycodone (15 mg: 22.6 ± 28.3; 30 mg: 35.2 ± 35.0) than with placebo, with significantly higher median scores with oxycodone (15 mg, *P* < 0.05; 30 mg, *P* < 0.0001; Table 4). Mean (±S.D.) E_max_ scores were 45–70 points greater with eluxadoline (100 mg: 62.8 ± 38.7; 200 mg: 74.2 ± 32.0) compared with both placebos, with significant LS mean differences compared with placebo (*P* < 0.0001 for all pairwise comparisons). Similarly, mean scores were 30–50 points greater with eluxadoline than with oxycodone, with significant LS mean differences compared with oxycodone (*P* < 0.0001 for all pairwise comparisons except eluxadoline 100 mg compared with oxycodone 30 mg (*P* < 0.01; Table 4).

#### PD Additional Measures.

Good Effects VAS, High VAS, and Any Effects VAS E_max_ scores showed a significantly greater LS mean difference with oral oxycodone compared with placebo (*P* < 0.0001 for all pairwise comparisons; Supplemental Table 1). As with the Drug Liking VAS data, there was a significantly greater LS mean E_max_ score on these three measures with oral eluxadoline 300 mg and 1000 mg compared with placebo (*P* < 0.01 for all pairwise comparisons) but a significantly lower LS mean score than with oxycodone (*P* < 0.0001 for all pairwise comparisons).

With intranasal oxycodone, there was a significantly greater median difference in Good Effects VAS score, a significantly greater LS mean difference in High VAS score, and a significantly greater median difference in Any Effects VAS E_max_ score compared with placebo (*P* < 0.0001 for all pairwise comparisons; Supplemental Table 2). Good Effects VAS and High VAS E_max_ scores were significantly greater with intranasal eluxadoline compared with placebo (*P* < 0.01 for all pairwise comparisons) and were lower than with oxycodone (*P* < 0.01 for all pairwise comparisons). Any Effects VAS E_max_ scores were significantly greater with intranasal eluxadoline compared with both placebo controls (*P* < 0.0001 for all pairwise comparisons), and were statistically lower than with oxycodone only for eluxadoline 100 and 200 mg compared with oxycodone 30 mg (*P* < 0.05 for both pairwise comparisons).

Alertness/Drowsiness VAS E_min_ scores were significantly lower with oral (median difference) and intranasal (LS mean difference) oxycodone compared with placebo (*P* < 0.01 for all pairwise comparisons), indicating drowsiness with oxycodone (Supplemental Table 1). Scores were not significantly different from placebo with oral eluxadoline but were significantly lower compared with placebo with intranasal eluxadoline (*P* < 0.001 for all pairwise comparisons; Supplemental Table 2). Scores with oral and intranasal eluxadoline were similar to and significantly greater than (indicating less drowsiness) those with oxycodone, respectively.

Drug Similarity VAS mean scores for codeine/morphine, heroin, benzodiazepines, and pseudoephedrine were higher with oral and intranasal oxycodone compared with placebo (Supplemental Table 3). Mean scores for oral and intranasal eluxadoline were higher than with placebo for all of these scales, particularly the codeine/morphine scale but remained low compared with those for oxycodone.

#### PD Objective Measures, Pupillometry.

**Fig. 3.**
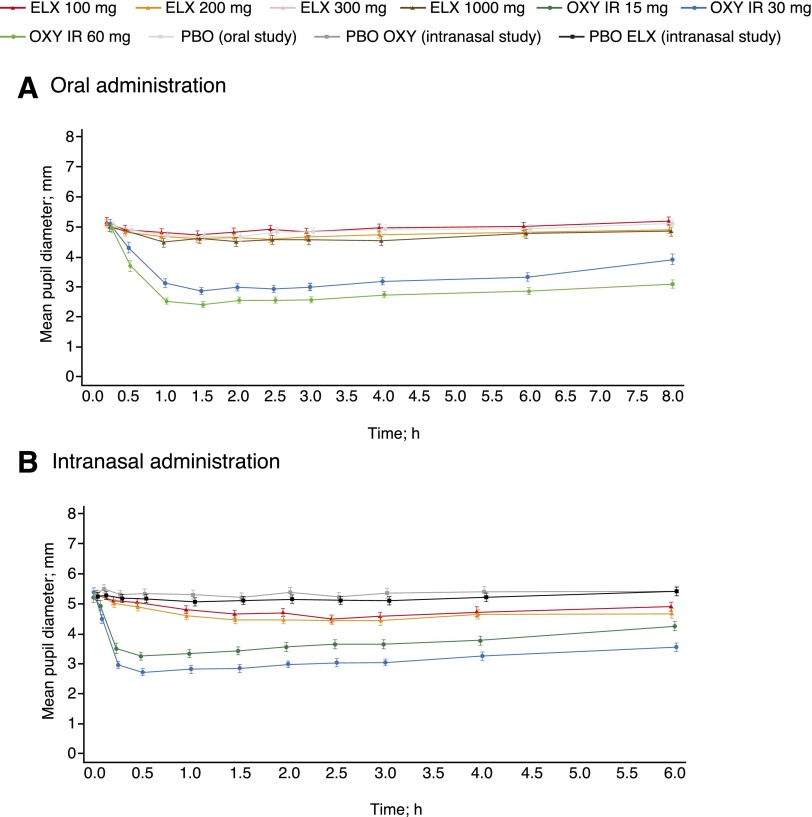
Mean pupil diameter over time following (A) oral or (B) intranasal drug administration. PD analysis sets. Data presented as mean ±S.E. ELX, eluxadoline; IR, immediate release; OXY, oxycodone; PBO, placebo.

Mean pupil diameter with oral oxycodone decreased significantly over time, reaching a minimum diameter at around 1.5 hours postdose and remaining lower than placebo throughout the sampling duration (up to 8 hours postdose), with a significant median difference in pupil diameter with both doses of oxycodone compared with placebo (*P* < 0.0001 for both pairwise comparisons; Fig. 3A and Table 3). By contrast, following oral eluxadoline administration, mean pupil diameter remained consistent for all doses and was similar to placebo, with little change from baseline, and no significant median difference were observed compared with placebo.

Following intranasal administration, mean pupil diameter reduced considerably with both doses of oxycodone, peaking at around 0.5 hours postdose and persisting throughout the sampling duration (up to 6 hours postdose), with a significant median difference compared with placebo (*P* < 0.0001 for all pairwise comparisons; Fig. 3B and Table 4). Mean pupil diameter decreased slightly with eluxadoline 100 and 200 mg compared with both placebo controls from approximately 1 hour postdose, with a significant median difference for both doses compared with both placebo controls (*P* < 0.0001 for all pairwise comparisons). However, the median difference was still significantly greater with oxycodone compared with eluxadoline (*P* < 0.0001 for all pairwise comparisons).

#### PD Nasal Effects.

Mean observer-rated nasal effects scale E_max_ scores were generally similar across treatments for nasal congestion, nasal irritation, and nasal discharge; however, nasal irritation was significantly greater with both doses of oxycodone compared with placebo and both eluxadoline doses (Supplemental Table 4). The mean percentage of dose insufflated (±S.D.) in the intranasal study was 55.0 ± 36.9 for eluxadoline 100 mg and 50.9 ± 40.1 for eluxadoline 200 mg, compared with 91.4 ± 20.9 for oxycodone 15 mg, 96.2 ± 13.1 for oxycodone 30 mg, 85.7 ± 28.2 for placebo lactose, and 64.9 ± 32.6 for placebo eluxadoline.

Mean scores for subject-rated nasal effects were similar across treatments for the burning subscale, were slightly higher with eluxadoline 100 and 200 mg and placebo eluxadoline for need to blow nose and runny nose/nasal discharge, and were considerably higher for eluxadoline 100 and 200 mg for facial pain/pressure and nasal congestion.

#### PK.

Mean maximum observed eluxadoline plasma concentrations (C_max_; ng/ml; ±S.D.) following oral administration increased with increasing dose (eluxadoline 100 mg: 2.3 ± 2.3; 300 mg: 7.6 ± 6.5; 1000 mg: 23.8 ± 17.8); median time to C_max_ [time to maximum observed plasma concentration (T_max_)] was 2.08 hours for eluxadoline 100 mg, 2.07 hours for eluxadoline 300 mg, and 1.08 hours for eluxadoline 1000 mg. Mean C_max_ following intranasal administration also increased with increasing dose (eluxadoline 100 mg: 118.8 ± 100.5; 200 mg: 191.4 ± 167.3), with peak concentrations being 5- to 8-fold greater than those seen with the highest dose of oral eluxadoline. Median T_max_ was 0.33 hours for eluxadoline 100 mg and 0.35 hours for eluxadoline 200 mg following intranasal administration.

#### AEs.

AEs occurring in ≥10% of participants with onset during the treatment phase are reported for both studies (Supplemental Table 5). Of the AEs related to abuse potential (euphoric mood, somnolence, dizziness, fatigue), somnolence and fatigue occurred at similar frequencies with eluxadoline and oxycodone, whereas dizziness was more common with oxycodone. Euphoric mood was markedly more common with both oral (30 mg: 75.7%; 60 mg: 73.0%) and intranasal (15 mg: 43.8%; 30 mg: 65.6%) oxycodone than for oral (100 mg: 14.3%; 300 mg: 19.4%; 1000 mg: 27.8%) or intranasal (100 mg: 21.9%; 200 mg: 18.8%) eluxadoline.

## Discussion

These studies examined the subjective and objective effects of oral and intranasal eluxadoline versus placebo and the active control oxycodone, in recreational, nondependent opioid users. The primary endpoint, Drug Liking VAS E_max_, showed minimal differences from placebo following oral eluxadoline administration, with statistically significant differences observed only at supratherapeutic doses (300 and 1000 mg). By contrast, E_max_ scores with oral oxycodone were significantly greater than with eluxadoline and placebo. Despite achievement of high systemic levels following intranasal eluxadoline administration, with peak scores 5- to 8-fold greater than with the highest oral eluxadoline dose, E_max_ scores were near neutral for intranasal eluxadoline, indicating that E_max_ scores do not correlate with systemic exposures. E_max_ scores for intranasal oxycodone were significantly greater than with eluxadoline and placebo. Drug Liking E_min_ scores revealed significant disliking of intranasal eluxadoline versus placebo and oxycodone. Along with results from additional secondary subjective measures, these data indicate that oral and intranasal eluxadoline are generally similar to placebo or disliked versus placebo. Negative effects were more prominent than the minimal positive effects with eluxadoline, as evident in responses to next-day measures, with participants showing no willingness to take oral or intranasal eluxadoline again. AEs of euphoric mood were observed with both oral and intranasal eluxadoline at a much lower frequency than with oxycodone.

The overall study design was consistent with the US Food and Drug Administration draft guidelines (http://www.fda.gov/downloads/drugs/guidancecomplianceregulatoryinformation/guidances/ucm334743.pdf). The qualification phase ensured that enrolled participants could discriminate between oral or intranasal oxycodone and placebo. During the treatment phase, significant differences were observed in both studies with the positive control, oxycodone, versus placebo in the primary endpoint (Drug Liking VAS E_max_) and other secondary endpoints, indicating the validity of the study approaches and sensitivity of the study measures. Additionally, the mean and range of peak responses following oxycodone administration were consistent with findings in other oral and intranasal studies of nondependent recreational opioid users (Walsh et al., 2008; Zacny and Lichtor, 2008; Perrino et al., 2012).

The results of these studies should, however, be interpreted in light of certain limitations. Participants in the intranasal study were able to insufflate only around 50% of the total drug quantity of eluxadoline, and the maximum dose was restricted by the bulk of the tablets, with 100 mg of eluxadoline being contained in an 824-mg tablet. However, difficulties in insufflation and the adverse nasal effects experienced suggest that maximum abusable eluxadoline doses were evaluated in this study, as it would be challenging to self-administer greater quantities to achieve a greater effect.

The DEA has ruled that eluxadoline be placed into Schedule IV of the Controlled Substances Act, defined as drugs with a low potential for abuse and low risk of dependence (US DEA, 2015a). The ruling stated that eluxadoline has a low abuse potential compared with drugs in Schedule III, may lead to only limited psychologic dependence, and has a currently accepted and approved medical use in the US and addresses an area of unmet need (US DEA, 2015a). The DEA considered that eluxadoline possesses a similar abuse potential to other drugs in Schedule IV, such as the relatively nonselective *μ*- and *κ*-OR ligand pentazocine, and the *μ*- and *κ*-OR ligand butorphanol, on the basis of their pharmacological similarities and data on the relative abuse potential of these drugs (Schlaepfer et al., 1998; Zacny et al., 1998; Preston and Bigelow, 2000; US DEA, 2015a). However, clinical studies are a proxy measure of a drug’s abuse potential, and epidemiologic data will be required to determine whether eluxadoline is in fact subject to abuse. The peripheral *μ*-OR agonist loperamide, an antidiarrheal, showed evidence of limited abuse potential in clinical studies (Jaffe et al., 1980) but is not a drug of abuse. Furthermore, the mixed pharmacological profile of eluxadoline may attenuate its abuse potential, consistent with studies of selective *δ*-OR antagonism in mice (Abdelhamid et al., 1991; Schiller et al., 1999). Additional data from human drug discrimination or self-administration studies could provide further information on the abuse potential of eluxadoline.

Several factors have contributed to this scheduling decision, including preclinical animal data, data from the current human abuse potential studies, and certain adverse events observed in phases 1 to 3 studies. The preclinical data suggest that oral eluxadoline is not associated with any significant systemic exposure or neurobehavioral effects. Eluxadoline was mostly below the lower limits of detection in jugular vein blood in rats following oral gavage (10 mg/kg), and below the limits of detection in the blood at all time points from 0.25 to 24 hours postdosing in cynomolgus monkeys given a single oral dose (5 mg/kg) of eluxadoline (Wade et al., 2012). Rats treated with eluxadoline at doses up to 300 mg/kg by oral gavage did not show any behavioral changes, and cynomolgus monkeys treated for 14 days with oral eluxadoline up to 20 mg/kg per day showed only limited behavioral changes. In a longer study, cynomolgus monkeys treated with eluxadoline up to 200 mg/kg per day via oral gavage showed no behavioral changes at any dose throughout the 9-month treatment period. Additionally, behaviors indicative of withdrawal were not observed in the recovery periods following chronic oral administration (US DEA, 2015b).

In preclinical studies, eluxadoline showed generalization to morphine and was self-administered to a greater extent than saline in heroin-trained monkeys. However, owing to interspecies differences, the relative exposure in animals at the lowest positive dose (3.2 mg/kg) was 170- an0d 1000-fold higher, respectively, than with the highest intranasal and oral doses in the present study, as the maximum oral or intranasal eluxadoline dose that could be administered was limited. Additionally, the intravenous administration in the preclinical studies may result in earlier peak exposure and onset of effect versus oral or intranasal administration, and may contribute to reinforcement potential (Abreu et al., 2001; Samaha et al., 2004). Previous animal studies of intravenous administration of antidepressants have shown positive results, whereas abuse potential studies in humans, typically conducted with the intended route of administration, demonstrated a reduced or no signal (Lamb and Griffiths, 1990; Tella et al., 1997).

The current human abuse potential studies with oral and intranasal eluxadoline revealed minimal differences versus placebo in certain subjective measures that were small in magnitude but statistically significant. Intranasal eluxadoline was associated with significant disliking versus placebo and oxycodone on a number of subjective measures. Since intranasal placebo eluxadoline also showed a similar frequency of AEs related to nasal effects as eluxadoline, disliking of eluxadoline may not be solely the result these effects and may instead be centrally mediated, suggesting that intranasal eluxadoline has inherent aversive properties. Such properties could be attributable to the *κ*-OR agonism of eluxadoline, as *κ*-OR agonists have been characterized by AEs such as dysphoria, detachment, and subjective Bad Effects (Schlaepfer et al., 1998; Zacny et al., 1998; Walsh et al., 2001; Chavkin, 2011). Although AEs of euphoric mood were observed with oral and intranasal eluxadoline, these were approximately 5-fold less frequent with the therapeutic dose of eluxadoline (100 mg) versus oxycodone when administered orally, and approximately 3-fold less frequent when administered intranasally. Furthermore, the lack of miosis induced with oral eluxadoline, even at 10 times the approved therapeutic dose, suggests that the occurrence of these AEs in this population of recreational drug users may represent an anticipatory response to receiving a drug, as opposed to a centrally mediated effect, and may suggest the lower rates of abuse-related AEs in the phase 3 trials are more representative of the true incidence of these events.

Additionally, the rates of these AEs are inconsistent with the results of two large phase 3 trials in IBS-D patients, in which events of euphoric mood were observed in two patients (0.2%) with eluxadoline 100 mg, and events of feeling drunk were observed in two patients (0.2%), one with eluxadoline 75 mg and one with eluxadoline 100 mg (Lembo et al., 2016). A further analysis of abuse-related AEs and potential opiate withdrawal AEs following treatment termination in the clinical development program revealed no differences from placebo in the frequency of these events. Similarly, analyses using the Subjective Opiate Withdrawal Scale during the phase 3 program revealed no differences from placebo (Fant et al., manuscript submitted to *Therap Adv Gastroenterol*).

Overall, these studies demonstrate that supratherapeutic doses of oral or intranasal eluxadoline produced outcomes not comparable to those seen with the *μ*-agonist oxycodone (positive control), at the same time acknowledging that, despite markedly lower systemic exposures, supratherapeutic doses of oral eluxadoline produced small but significant differences from placebo in certain positive effects measures. Importantly, however, the therapeutic dose of eluxadoline was similar to placebo following oral administration on most subjective measures, and intranasal eluxadoline was generally associated with significant disliking versus placebo and oxycodone. Participants showed no willingness to take oral or intranasal eluxadoline again. AEs of euphoric mood were observed with oral and intranasal eluxadoline but were far less common than with oxycodone and may not be a centrally mediated effect. The current data demonstrate that eluxadoline has a lower abuse potential than other Schedule II *μ*-OR agonists in recreational opioid users.

## Abbreviations

AEs: adverse events
ARCI: Addiction Research Center Inventory
C_max_: maximum observed plasma concentration
DEA: Drug Enforcement Agency
E_max_: peak (maximum) effect
E_min_: peak (minimum) effect
IBS-D: irritable bowel syndrome with diarrhea
LS: least squares
OR: opioid receptor
PD: pharmacodynamic
PK: pharmacokinetic
VAS: Visual Analog Scale
